# Butterflies and Ribbons: Supporting Families Experiencing Perinatal Loss in Multiple Gestation

**DOI:** 10.3390/children10081407

**Published:** 2023-08-18

**Authors:** Béatrice Boutillier, Nicholas D. Embleton, Sophie Bélanger, Alexie Bigras-Mercier, Audrey Larone Juneau, Keith J. Barrington, Annie Janvier

**Affiliations:** 1 Division of Neonatology, CHU Sainte-Justine, Montréal, QC H3T 1C5, Canada; beatrice.boutillier@umontreal.ca (B.B.); sophie.belanger.hsj@ssss.gouv.qc.ca (S.B.); alexie.bigrasm@gmail.com (A.B.-M.); audrey.larone.juneau.hsj@ssss.gouv.qc.ca (A.L.J.); keith.barrington@umontreal.ca (K.J.B.); 2 CHU Sainte-Justine Research Center, Montréal, QC H3T 1C5, Canada; 3 Department of Medicine, Newcastle University, Newcastle NE1 7RU, UK; nicholas.embleton@newcastle.ac.uk; 4 Department of Medicine, University of Montreal, Montréal, QC H3T 1J4, Canada; 5 Unité D’éthique Clinique, Unité de Soins Palliatifs, Bureau du Partenariat Patients—Familles-Soignants, CHU Sainte-Justine, Montréal, QC H3T 1C5, Canada; 6 Clinical Ethics Unit and Palliative Care Unit, Department of Pediatrics and Clinical Ethics, University of Montreal Neonatologist, Sainte-Justine Hospital, 3175 Chemin de la Côte-Sainte-Catherine, Montreal, QC H3T 1C5, Canada

**Keywords:** multiple pregnancy, twins, triplets, neonatology, palliative care, prematurity, communication, parental perspectives, medical education, perinatal loss, nursing

## Abstract

**Introduction:** In neonatology, multiple pregnancies are common. Unfortunately, it is not rare for one baby to die. Communication with parents in these circumstances has been demonstrated to be sub-optimal. **Methods:** Two educational programs were evaluated with pre- and post-course surveys, questionnaires administered to participants, and audits. **Results:** In the online Butterfly project (UK; n = 734 participants), all participants reported that the training exceeded or met their expectations, 97% reported they learned new skills, and 48% had already applied them. Participants expressed gratitude in their open-ended answers: “*I feel a lot more confident in supporting parents in this situation*”. In the Ribbon project (workshop for neonatal clinicians, Quebec; n = 242), 97% were satisfied with the training and reported feeling more comfortable caring for bereaved parents. Knowledge improved pre–post training. Audits revealed that 100% of cases were identified on the incubator and the baby’s/babies’ admission card, all changed rooms after the death of their co-twin/triplet, and all had the name of their co-twin/triplet on the discharge summary. All clinicians (55) knew what the ribbon symbol meant when asked during surprise audits at the bedside. **Conclusion:** Different educational strategies to optimize communication with families after the perinatal loss of a co-twin are appreciated and have a positive impact.

## 1. Introduction

Parents may suffer reproductive losses at different stages, leading to miscarriage, stillbirth, or neonatal death. Many bereaved parents undergo complex patterns of grief which can involve sadness, anxiety, guilt, and anger [1]. The grieving process may lead to mental health problems [2,3]. Perinatal loss is more strongly associated with complicated grief than other forms of loss [4,5]. Perinatal loss occurs more frequently in multiple pregnancy (twins, triplets, or higher order), in which case the process can be more complex as fetuses may die at different stages of pregnancy or in the neonatal period, and the remaining sibling(s) may survive [4]. Multiple gestations increase all pregnancy risks, such as diabetes, high blood pressure, incidence of operative delivery, transfusions, etc. Multiple gestations also increase the risks to children, including mortality. Parents can experience the death of a child who is a twin, triplet, or higher order multiple at several time points. Some children die in utero from placental or cord malfunction or accidents, or in some cases because of a selective termination of pregnancy. Children can also die at birth from unsuccessful resuscitation if they are extremely preterm and/or if they have a significant anomaly. About half the twins and almost all triplets are admitted to a neonatal intensive care unit (NICU) because of prematurity. Prematurity is associated with an increased risk of mortality and morbidity. In many countries, the rate of multiple pregnancies has been increasing, and the loss of a baby from a multiple pregnancy is thus observed more often. Grief can be especially complex in such situations. Indeed, parents cannot avoid the hospital where their child died as they need to continue caring for the surviving sibling(s) while they mourn [4,6,7]. This is most commonly faced in the NICU when one of the children may have died prenatally, at birth, or in the NICU after a period of neonatal intensive care. There is an increased rate of prematurity after in utero demise of a fetus in a multiple pregnancy, and congenital anomalies, which may be life-limiting, are also more common in multiple births and are often discordant. There are few resources for parents in this situation [8].

**Figure 1:**
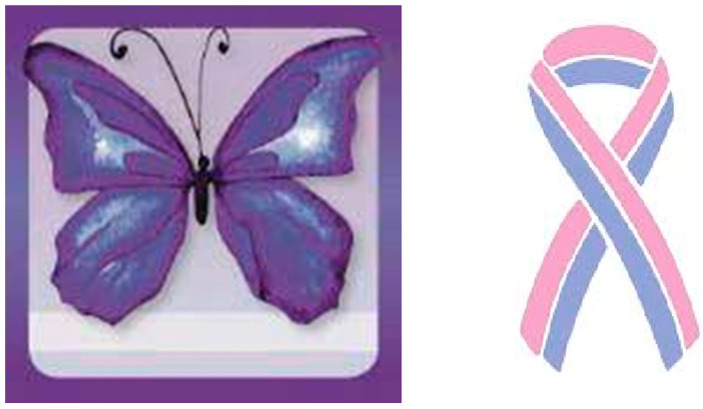
The symbols used to identify the loss of a co-twin of a triplet.

Interdisciplinary investigations have examined the views and experiences of parents suffering the loss of a co-twin or triplet, as well as a diverse group of health professionals including nurses, doctors, and midwives as well as health professionals working in the community [9,10]. These studies have shown that bereaved parents do not feel well supported and even feel abandoned for a number of reasons. First, many clinicians are unaware that they have suffered a loss and may ask questions such as “Do you have other children?”, often addressed to parents who suffered the loss days ago, in the same unit. Second, when aware, many do not know what to say or how to support a family. They tend to only focus their attention on the surviving child(ren). The situation of these parents is unique in the healthcare system: they suffered the loss of a child and are grieving, but at the same time, one baby is still alive and often making progress. They must continue to visit the hospital where many feel they have experienced a trauma and try to celebrate the achievements of their surviving baby(ies). General education in ethics and palliative care is limited in the medical and nursing curriculum, especially perinatal palliative care. Education about this very specific and unique situation in palliative care (that is still quite common in NICUs) is generally absent from teaching curriculums. Third, when trying to help, some comments or actions may be harmful, such as “*At least you have another one*” [9,10]. After these multiple studies, a checklist was developed to optimize the care of these families [9,10]. Eight themes and steps important to bereaved parents were to be incorporated into training, education, and practice, and were co-designed by parents and staff (Table 1). These themes were validated in a series of workshops with parents, the public, advocacy groups, and health professionals [11]. During the workshops, a parent in Newcastle suggested that a butterfly symbol, to identify a baby whose co-multiple(s) had died, could be placed on the incubator or cot of any surviving baby (Figure 1). The butterfly project, aimed at teaching those eight important themes, was born, developed, and attracted international interest.

The Ribbon project was developed at the CHU (University Health Center) Sainte-Justine for the needs of clinicians and parents in Montreal (the largest level 4 NICU in Canada, where the parents are almost all francophone). This teaching is also based on the eight themes developed using extensive parental perspectives. The parent advisory board did not appreciate the butterfly symbol to represent perinatal and neonatal deaths. They preferred to avoid the butterfly symbolism and desired a neutral symbol, choosing the perinatal loss ribbon as a symbol (Figure 1). These programs are the results of these studies and reflections on the perinatal death of babies from multiple pregnancies. Their goal is to train clinicians to support parents in these complex situations that are not rare in NICUs.

The eight key themes which are important to many parents of multiples who have experienced perinatal loss (Table 1) form the basis of these two training programs. The goal of this article is to examine the perspective of clinician participants and the impacts of these teaching programs. A secondary goal is to optimize knowledge transfer and teaching about this topic in obstetrics, neonatology, and pediatrics. For this reason, the PowerPoint presentation is available (Supplementary Material) and can be used by clinicians who meet bereaved parents in these difficult situations.

### 1.1. The Butterfly Project

As stated in the introduction, the Butterfly project began in Newcastle, UK, in 2016 with the goal of improving support for parents with multiple pregnancies experiencing perinatal loss, but with one or more infants admitted to the neonatal intensive care unit (NICU). This teaching initiative was based on extensive research on the perspectives of bereaved parents [9]. Parents reported on eight themes that were important to them (Table 1). Our goal was to develop a training program to teach those themes and optimize the communication of clinicians. Another investigation, with staff caring for surviving babies, revealed their discomfort in dealing with the situation and how we could help them [10]. We gained further funding for a film-based project where we presented the themes developed from those projects. We then created cot cards and medical chart stickers using a butterfly symbol (Figure 1) to alert staff to the situation. The butterfly concept was shared across health networks and has been adopted by many hospitals worldwide, and supported by co-designed information leaflets, “tips” for families, friends, and staff, along with PowerPoint slides, as well as practice guidelines, which have now been translated into more than 15 languages. Films and resources were made free to access. The www.neonatalbutterflyproject.org (accessed on 14 August 2023) has been viewed over 10,000 times globally.

Finally, we created a massive open online course (MOOC) enabling free global access [12]. This 4 h course gained accreditation from professional organizations for CME/CNE (continuing medical/nursing education) and has been endorsed by more than 20 advocacy organizations worldwide. This course is designed for clinicians, but a small number of parents have also completed the online training. Participants in the course are informed that they will be asked for feedback as part of a continuous quality improvement initiative. At the end of the course, participants in the program are asked to provide feedback using an online form asking three questions about the course: “Did it meet expectations?”, “Did you gain new knowledge or skills?”, and “Have you applied what you learned”? There is also an opportunity to leave comments.

### 1.2. The Ribbon Project

At the CHU (university health center) Sainte Justine, a level 4 mother–child hospital (67-bed NICU and 1000 admissions a year), we developed a similar course adapted from the Butterfly project. We use a ribbon symbol, chosen by the NICU’s bereaved parent advisory group. The training was developed as a 1 h course for all NICU clinicians. The course was accredited, free of charge, and is now available online. The training was designed to address the essential themes identified by the research described above (Supplementary Material: PowerPoint presentation, English and French versions).

Participating clinicians in the Ribbon program were surveyed before and after course completion to assess their comfort level and knowledge: if they felt they had gained knowledge and were comfortable dealing with parents in this situation. They were also given a vignette addressing the death of one of the preterm twins and asked specific questions about their approach. Clinicians were informed this was a quality improvement study to improve the care we provide to parents of bereaved twins/triplets. They agreed to participate in this project when they attended the training, which includes pre- and post-training questions. Answering the pre- and post-training questionnaire indicated that they were willing to participate in the quality improvement study. They were also informed we would perform audits to investigate whether what they were taught was applied in a clinical setting. We performed four unannounced audits in the NICU of CHU Sainte Justine to determine the level of adhesion to the program. During these audits, we examined (1) whether the identifying symbol with the name of the deceased baby was being used (on the baby’s identification card, the hospital chart, and the baby’s incubator), (2) whether the surviving baby changed rooms when his/her twin died, (3) if the name of the deceased twin was present in the discharge summary, and if the information was relayed to physicians in the community. We also asked the clinicians of the surviving baby/babies at the moment of the audit what the ribbon meant and if they knew how to communicate with families about the loss of a co-twin/triplet (if they did not, we asked them if they knew who could). During the first two audits in 2019 and 2020, we also asked the parents of surviving twins/triplets if they were informed of the quality improvement program that aimed at improving the care for families in their situation. They were asked if they wanted to give their (confidential) opinion about the program, whether they were satisfied, and if they had any comments. Parents were not considered to be research participants.

## 2. Statistics

Descriptive statistics were used to report participants’ perspectives and knowledge. Answers to the open-ended questions were analyzed using descriptive content analysis. Themes were developed simultaneously and independently by a team of three investigators. During the initial exploration of the data, codes, sub-codes, and coding definitions and structures were developed gradually until a consensus was reached. Excel was used to identify and report the frequency of themes invoked by the participants.

This quality improvement investigation was accepted by the CHU Sainte-Justine research center. Clinicians were informed that this was a quality improvement initiative and investigation. They were informed that they were free to answer the pre- and post-training questionnaires and participate in the clinical audits. They were informed that by answering the questionnaire, they agreed to participate in this quality improvement investigation.

## 3. Results

### 3.1. The Butterfly Project

Since the launch of the online course in November 2021, 1370 learners from over 90 countries have enrolled including doctors, nurses, midwives, psychotherapists, ultra-sonographers, psychologists, and parents; 734 have provided feedback. Of those, more than 90% stated that the course had changed their clinical practice and that of others in their teams; 100% of participants reported that the training exceeded or met their expectations; 97% reported that they learned new skills, and 48% had already applied them in practice.

The themes of gratitude, empowerment, and knowledge were the main ones invoked by participants in their comments. Indeed, participants described having more confidence to support bereaved parents, for example: “*I feel a lot more confident in supporting parents*” and “*I feel prepared, capable of listening with empathy; I also give myself permission to feel moved and touched by the stories I hear*”. Other themes included the practical usefulness of the course, and the wish to share their skills with other clinicians, as well as the powerful emotions they were experiencing (and were better equipped to experience, and to self-reflect on their role), for example:

“*I found this course incredibly useful for my future practice.*”

“*This course has been very powerful, emotive and thought-provoking. I have shared it with colleagues, home and abroad.*”

“*This is a fantastic course—thank you. I will definitely be sharing.*”

“*What an amazing resource to allow us as professionals to take a little time to understand, by the use of parent’s stories, how important the things we say and do make such a big difference.*”

“*This course is outstanding, so much information to help others and raise awareness. Before this course I knew next to nothing, coming to the end of this course I feel more confident going out there and supporting these parents.*”

While this course is offered to optimize the communication and care clinicians provide to bereaved parents, some parents also participated in the training program and gave positive comments.

### 3.2. The Ribbon Project

The course was delivered to clinicians in a large level 4 NICU in Quebec. There were 242 participants, and the average age of participants was 31. The majority were nurses and women between 25 and 30 years old (Table 2). Before the course, 74% answered that they found it difficult to care for grieving parents in multiple pregnancies. In the post-course questionnaire, 97% were satisfied or very satisfied with the training and 97% reported feeling more comfortable caring for bereaved parents in this situation.

### 3.3. Knowledge Improved Pre–Post Training

In the pre-training questionnaire: 28% of the caregivers thought that the number next to the other twin’s name (used to identify the order of delivery in a multiple pregnancy) should be removed without talking to the parents, 8% avoided talking about the deceased twin, and 41% were not comfortable talking about it. A total of 6% of the professionals felt unprepared to mention the subsequent follow-up or the help that the local health center could provide.

Post-training: 183 participants responded to the post-training questionnaire; all respondents recognized the importance of sensitizing caregivers to the bereavement situation, less than 1% said they would avoid talking about the deceased twin, and only 3% reported being uncomfortable with it. Only 4% thought it is a good idea to remove the twin sequence number from the patient identification cards, or did not know what to do.

### 3.4. Clinical Audits

In four clinical audits performed between 2019 and 2022, 100% of cases were identified (28/28; 23 twins and 5 triplets surviving; 8 antenatal deaths) with the ribbon sticker on the incubator and also on the identification card and the hospital chart, and 100% of the surviving twins/triplets kept their identification (#1, 2, 3) after it had been verified that it was the parents’ wish. All the infants who were in a double or twin room were moved to a single-patient room after the death of their co-twin-triplet, all had the name of their co-twin/triplet on the discharge summary and the bereavement was noted. All clinicians questioned (n = 55), including nurses, respiratory therapists, medical trainees, and consultants, knew what the ribbon symbol meant; 38/55 (71%) stated that they felt comfortable dealing with parents in this situation and for those who did not, they knew who to ask for help.

During the first two audits, all the parents (n = 10) who were at the bedside knew what the ribbon project was because clinicians had shared the goal of the program with them. They knew the meaning of the ribbon(s) next to their baby’s/babies’ name(s) on the incubator. On the one hand, parents revealed the suffering generated by ignorance or negation of the situation that they experienced outside of the hospital with their relatives or friends. On the other, they reported gratitude and the importance of feeling cared for and supported in the NICU.

## 4. Discussion

The majority of clinicians in neonatology report a lack of knowledge and training on bereavement, including how to care for families and how to interact with them [13]. Even caregivers who deal frequently with the mortality of their patients, such as in intensive care and oncology, are often trained “on the job” and may not learn optimal practices [14]. In medicine, when death occurs, it is not rare for the clinicians present to only briefly be in contact with a family, and further bereavement support will take place elsewhere in the community. By contrast, in the context of a co-twin/triplet’s death, the situation is unique. Parents continue to be in contact with NICU clinicians and must continue investing in the care of, and keep hope for, the surviving sibling(s). This difficult situation may cause additional pathological grief [1,2]. It is already complex to grieve the loss of a baby, and it is also difficult to endure a long and sometimes tumultuous NICU hospitalization; doing both at the same time is a serious challenge. This difficulty is compounded by the lack of knowledge and comfort of healthcare professionals. Indeed, as described in the literature, caregivers may not even know that a baby is a surviving twin and ask questions that harm the parents, such as “Do you have other children?” (when the co-twin died days ago). In other cases, they may be aware of the death but are uncertain about how to act and, with the best of intentions, often act in ways contrary to the wishes of the parents, by failing to acknowledge the status of surviving twin, not speaking about him/her, or by minimizing their grief or failing to recall that the parents are bereaved [9,14]. Sometimes, they may even say very harmful sentences that they think will help, such as: “*At least you have another one*”.

In published literature concerning perinatal grief, parents often emphasize the importance of the recognition of their grief and the need for emotional and moral support from the health care team [9,14]. Most clinicians have not been trained to provide this support and do not feel comfortable doing so [10], and the need for targeted professional training in perinatal loss has been a frequent recommendation [13]. The participants’ evaluation of these courses in their responses to the open-ended questions show a great deal of satisfaction with the knowledge gained, but also the ability they had to self-reflect. In addition, one common aspect of these two training courses is the participation of bereaved parents who have agreed to share their stories and experiences, and the caregivers saw this as a great learning experience: “*I am so grateful to parents and clinicians for sharing their stories*”. The feedback from parents during the ribbon audit demonstrates the essential role that this training can play in the care of parents. This painful and tragic experience can be softened and made less difficult by the behavior of trained clinicians.

The discovery of a multiple pregnancy may lead to immediate feelings of celebration, as multiple gestation is often idealized and the risks minimized [15]. This is particularly the case when pregnancy results from medically assisted reproduction. This unique situation, in the case of the death of a twin, makes mourning more difficult, and phrases such as “*at least you have one left*”, that suggest that just having one baby is the “norm”, are all the more painful.

Clinicians, other family members, and/or loved ones may wish to concentrate on the positive, avoid speaking about the dead child, and end up minimizing parental grief. The training programs we describe here, which are rich in theory and developed from the experiences of parents, and which also include practical exercises, have been found by participants to be very beneficial for knowing how to practically help these parents and behave in their presence. Simple sentences that can be used are described and clinicians can use them in these situations. Parents may tend to focus on the living child while being unsure of how to grieve their dead baby in the NICU, [5,16,17] so it is crucial for clinicians to address the issues and support the parents through their complex grieving process. We have described two possible ways to teach clinicians. The Butterfly project can be completed by any clinician, anywhere, online, and takes about 4 h. Online courses were extremely helpful during COVID when clinicians could not gather in the same room for teaching. The Ribbon project (Supplementary Material, including course material in French) takes an hour and can be offered to obstetric, perinatal, and/or NICU clinicians. In many centers, teaching hours are limited and/or non-existent and may not even be funded. The Ribbon course can be offered in person (such as in this study) or online (we have started to offer it online to new clinicians). These materials can be adapted to the reality of different units, and can also be taught to a parent who has suffered the loss of a twin/triplet. Each unit is different, and clinicians interested in family partnership, ethics, and/or palliative care will often know how to adapt the training to their own unit needs. These studies have several limitations. They are based on questionnaires, which have their inherent limitations. While we performed clinical audits, we did not formally examine the psychological impacts of this training on families. On the other hand, this is the first investigation describing training programs based on the needs of parents.

Clinicians find these situations very difficult but appreciate the training they have received. Neonatology is challenging for clinicians, who report a frequent occurrence of moral distress [18]. In previous studies, it was found that any intervention which increases caregivers’ sense of achievement, satisfaction, and appreciation that they have responded to a family’s needs will likely reduce their distress [19]. Many parents in this study have experienced interactions with trained caregivers which can be described as best practice, where, with empathy and insight, the caregivers recognized the simultaneous grief and hope of parents who have lost a co-twin/triplet. These parents were grateful and felt that their relationship with the clinicians was strengthened.

This study strongly suggests that formal training programs for clinicians in NICUs, using either the Butterfly or Ribbon approaches, should be routinely offered. Both formats (online and in person) and lengths of the program (1 h or 4 h) are considered useful and translated into changes in practice. The care for families in this difficult situation can be improved, while clinicians feel empowered to provide optimal care.

## Figures and Tables

**Table 1:** Key themes covered in the Butterfly and Ribbon projects.

Identify and recognize twin status Acknowledge the bereavement Support the parents emotionally Inform: provide appropriate information Continuity of staffing and information Memory making: physical, emotional Logistics: cot occupancy and position in the NICU Discharge: prepare parents for going home

**Table 2:** Demographic information of participants in the Ribbon project.

	Total	242	%
Sex	Female	228	94%
	Male	14	6%
Age (years)	20–35	143	59%
	36–55	92	38%
	56+	7	2%
Experience (years)	0–2	98	39%
	3–10	81	32%
	10 and +	63	25%
Profession	Nurse practitioners	3	1%
	Nurses	162	67%
	Auxiliary nurses	32	13%
	Respiratory therapists	18	7%
	Neonatologists	1	0%
	Social workers	2	1%
	Others	23	9%
Shift	Day	109	45%
	Evening	55	22%
	Night	79	32%
First training about the subject		225	93%
Has already treated a child whose twin died		184	76%

## Data Availability

Data is not available for this article, but authors can be contacted for more information.

## Supplementary Materials

The following supporting information can be downloaded at: https://www.mdpi.com/article/10.3390/children10081407/s1.

## Abbreviations

Neonatal intensive care unit (NICU)
