# Supplementary material for: Butterflies and Ribbons: Supporting Families Experiencing Perinatal Loss in Multiple Gestation
Source: Children (Basel). 2023 Aug 18;10(8):1407. doi: 10.3390/children10081407 (PMC10453894; doi:10.3390/children10081407)

SUPPLEMENTAL MATERIAL

-Ribbon course material in English

-Course material in French

CHU Sainte-Justine
Le centre hospitalier universitaire mère-enfant
Université de Montréal

RQSPAL
RÉSEAU QUÉBÉCOIS DE RECHERCHE EN SOINS PALLIATIFS ET DE FIN DE VIE

The Ribbon Project:

Perinatal grief in multiple pregnancies

Alexie Bigras-Mercier BNurs., Sophie Bélanger NNP, Keith J Barrington MB ChB, Annie Janvier, MD PhD, N Embleton MD.

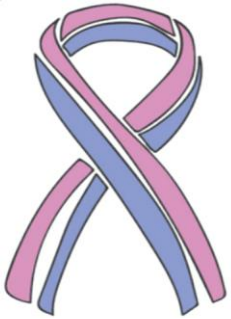

Introduction

- Twins and triplets: significant proportion of NICU admissions
- Mortality of one baby is not rare
- Often, the parents and clinicians are the only ones to have met the babies
- Parents feel grief at same time as trying to care for the surviving baby(s)
- Clinicians often are uncertain how to address this difficult situation
    - When unaware of the death, we can unintentionally say harmful things “*do you have other children*?”
    - Harmful comments: “at least you still have one baby”
    - Do we talk about the baby who died? How?

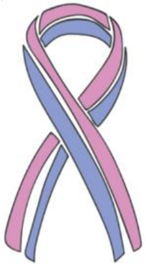

Plan for today
Nick Embleton and the butterfly project
www.neonatalbutterflyproject.org

- Dr Nick Embleton and his team have investigated this topic
- This presentation is designed for NICU and perinatal clinicians, is an hour long and answers the following questions:
    - How do parents want us to care for them, what do they need? What are their expériences?
    - How can we train clinicians better to deal with these complex situations?
    - What can we do practically to help parents?

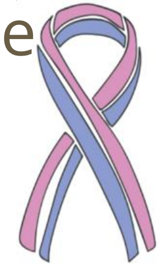

Introduction

- Clinicians often have a lack of training/confidence when dealing with bereaved parents in these situations
  - how to start a conversation about the loss?
- Parents expect and value care form clinicians
  - Clinicians are often the only ones to have ‘known’ the deceased baby
- The way in which clinicians relate to parents has a major impact on their experience
  - *"There was one or two who were kind of cold to the counselling part and those aren't the ones you favoured, because they are there to do a job, not for you"* [father]

Important Themes identified by parents:

Richards et al. (2015). Mother's perspectives on the perinatal loss of a co-twin: a qualitative study. BMC Pregnancy and Childbirth, 15:143.

1. Recognition of the multiple pregnancy
2. Recognition of parents' grief
3. Offering emotional support
4. Providing relevant information
5. Assuring continuity of care and of information
6. Creation of souvenirs
7. Being sensitive to the location of babies in the NICU
8. Preparation for discharge

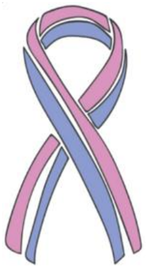

Recognise the twin status

‘Nurses would ask ‘How was your pregnancy with him?’ *They forget about him being a twin and everything like that‘* [Mother]

‘Clinicians would ask ‘do you have other children?’ *They did that to be nice. The question hurts. Well yes, he died a week ago here, with your team‘* [Father]

Recognise the twin status

‘they [staff] all acknowledged what’s happened, and I think that that was really important….nobody ever tried to treat us like parents who’d just had one baby’ [Mother]

‘..one of the senior nurses…when we were there and he mentioned the deceased twin… and he just came over to me and said ‘we know what you’ve been through…if you ever want to talk about it or you want any, you know what I mean, we are here, we will not ram it down your throat, but we are here’ [Mother]

Recognise the twin status

‘*they forget about him being a twin* [Father speaking]’ ‘*it’s not just they forget…a lot of them didn’t even know* [Mother]’

‘a little blue butterfly…….just put a butterfly on [the cot] *then they know he’s a twin and then it solves the problem’* [Mother]

Recognition of the multiple pregnancy

«They all knew what had happened, no-one treated us as if we had just one baby» [Mother]

«...they'd always write the name and then 'twin 1' or 'twin 2' underneath and, they asked us if we wanted that to be done or not done you know, so they were thinking about how[we felt]... I think what you didn't want was, you know, as soon as my other child had died was everyone just treating the surviving one as if he was a singleton, because he wasn't'.» [Mother]

The Ribbon Symbol

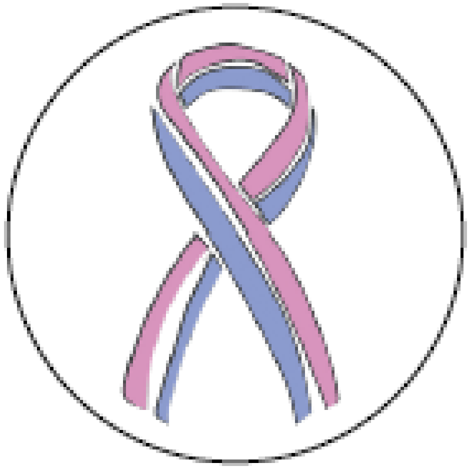

«They forgot he was a twin» [Père] «They didn't forget, they simply didn't know» [Mother]

«A little blue butterfly... a simple symbol on the incubator and then they all knew he was a twin» [Mother]

The Ribbon Symbol

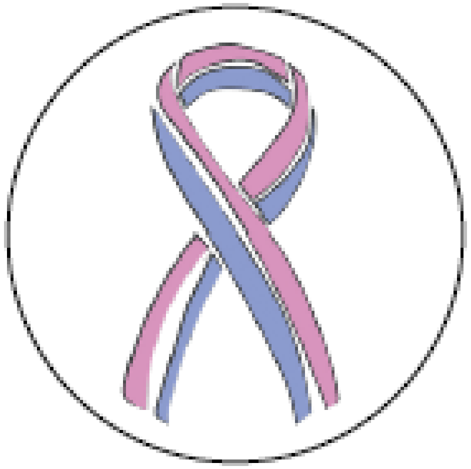

Our parent group preferred another symbol, as many did not like the symbol of the butterfly

The parent group chose the perinatal loss ribbon symbol. The name of the deceased baby is written on the ribbon.

The symbol is placed in many locations after the death of a twin or triplet: incubator, patient card, medical chart, consultations for specialists, nurses « kardex ».

Acknowledge the bereavement

'One of the doctors at the time really quite upset me and she often said to me "at least you've still got one"…..that was one of the worst things that anyone could possibly say ever.' [Mother]

Acknowledge the bereavement

Both clinicians and parents admitted that they often focused too quickly on the surviving baby(ies).

‘I always felt I mismanaged that situation emotionally for her because for me it was all about the 28-week baby that was alive.......I felt I gave the best practical care but I always felt I hadn't acknowledged the other baby’s death in the way that I should… We don't acknowledge the grief, we try and focus on the positive. We should perhaps be dealing with both the positive and the grief at the same time.’ [Midwife]

Acknowledging the bereavement: practical issues

- Do not remove the identifers if one baby dies (Baby Smith 2 is still baby Smith 2, Baby Jones C...)
- Identify the situation for everyone who comes by (Place a ribbon sticker with the name of the deceased baby on the incubator, the surviving baby's card, the hospital chart, consultation requests...)
- Inform parents of why the ribbon is placed there
- Make sure staff are informed, that it is a routine and part of baby transfers at shift change.
- This ensures that people avoid painful questions (such as « *do you have other children*? »)

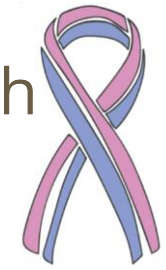

Ask parents

- Do they want us to continue to identify the baby as Bébé Lacaze 1?
- Tell them « we usually put a ribbon on the incubator and chart… »,
- We tell them what the ribbon is all about, and that we know it is very difficult to look after and celebrate the birth of the remaining baby, while still grieving the loss
- The ribbon is to help us remember
- Ask them if that is what they would want, and that it is also OK if they don't want it… « some parents don't want the ribbon »
- Ask if they had a name for the twin who died (if the death was quick and the name was not known to clinicians before the death).
- This is the first step (Acknowledge twin status, acknowledge bereavement) in providing support

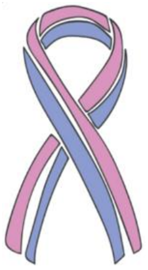

Death of co-fetus, is it different?

- It is also not rare for one child to die in utero
  - Twin-to twin transfusion syndrome, laser therapy
  - IUGR with perinatal death
  - Etc
- These cases can also be acknowledged: parents can be informed of the ribbon project and asked if they wish to have the ribbon with the name of the deceased twin.

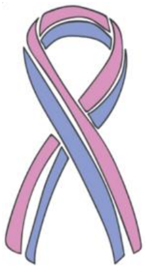

Provide emotional support (negative examples)

'it was just a bit matter of fact to people' [Mother - referring to the funeral of one of her twins]

'her middle name wasn't put in and it's smudged…' [Mother – referring to the remembrance book] *'and she just feels upset when she sees it'* [Father]

'one of the nurses that was least conscientious towards other people's feelings kept calling him [surviving twin] *by his brother's* [demised twin] *name'* [Mother]

Provide emotional support

‘ We appreciate that you really care, it isn’t just a job. If you are upset, that’s OK. ’[Father]

‘they [staff] weren’t allowed to get upset around you….you knew that they were kind of like leaving the room….a comfort that someone around you is upset with you….’ [Mother]

Emotional Support

- Includes organizing follow up with psychology, social workers, spiritual support
- Listening to parents and their needs
- Normalise their emotions

«It is normal to feel sadness and joy at the same time»

“Some parents want x others want y, many parents in this situation feel ___ the same way”

Provide appropriate information

‘ So all the way through ….we were totally informed, they didn’t try and hide anything…..you have to hear the honest truth but there is a way of putting it’ [Mother]

‘Things were bleaker than they said all at once right at the beginning and I think that’s definitely what we needed at the time…..they never lied…..’ [Mother]

Provide appropriate information

'She [nurse] talked us through you know the idea that we could get her baptised if we wanted to, we could see her, we could spend some sort of time with her....so she had told us all the options and then when it came to it on the day, they were really good...the other nurses... ' [Mother]

Provide appropriate information

‘We didn’t know where our dead baby was in the hospital. I didn’t have a clue where he was’ [Mother]

‘you’re 23 weeks into your pregnancy – I was thinking what am I actually going to give birth to, you know, is it going to look like a baby?’ [Mother]

Provide continuity

- *'It was, it was really good, we knew this nurse, she was there when our bby died, we trusted her sort of thing and they went off to get her quickly'* [Mother]

- *'A lot of days it was a new nurse....[meant] going through it a lot, that is hard.'* [Mother]

Offer memory making

- *‘The nurses did it, they took footprints and handprints; I’ve still not looked at them – I can’t….I’m pleased they did, but I don’t know if I’ll ever get to the stage that I could look at it’* [Mother]
- *‘I cherish all the memories in the box, the footprints, the clip of hair.’* [Mother]
- *‘I have got pictures….it’s nice to see that it was both of them together in the incubator.’* [Mother]

Handle bed placement sensitively

- *'It's a bit heartless putting us on a ward right next to parents and their* twins *just after the death'* [Mother]
- *'They were in a twin room and then one of them died and he got replaced by a new baby hours later. I know there are a lot of sick babies, but we could have been transferred to another room.'* [Father]
- *'One thing which I did find difficult ….I was put next to a lot of twins'* [Mother]
- *'It was really hard to see but you know it has to be done….you can't say you are never putting one in that cot ever again'* [Mother – talking about a new baby being put into the cot where her demised twin had been]

Prepare parents for discharge

'it was horrible going home when I had to come home that was really hard' [Mother]

'thought it would get better when I got home.... but it actually got a lot worse' [Mother]

'from a follow-up point of view I genuinely feel now that it's difficult to know who to turn to since I have home' [Mother]

Prepare parents for discharge

- Arrange a pre-discharge meeting with a member of staff (psychologist, social worker, consultant or nurse trained in neonatal loss)
- Offer bereavement support
- Put the parents in touch with a support group
- Provide contact details for appropriate groups
- Ensure the discharge summary includes the name of the deceased baby(ies)
- Arrange follow-up appointment for parents
- Provide reassurance about the health of the surviving twin

In summmary

-Ensure that all clinicians are informed of the multiple status

-Know the name of the deceased baby and use it. Don't avoid speaking about the deceased sibling

-Never minimize the loss (don't say, « *at least you still have one* »)

-Think carefully about incubator placement

-Create souvenirs

-Include the information in the discharge summary

-Ensure appropriate follow-up

In summmary

- Neonatal clinicians can do a lot to improve the experience for parents who suffer a loss with a multiple pregnancy
- There are some small things that can be done to make this experience less painful
- It’s important that staff give consideration to their own well-being too – accept your limitations and ask for help and support when you need it

Ressources

- MBF – The Multiple Birth Foundation http://www.multiplebirths.org.uk/
- CLIMB – Center for Loss in Multiple Birth http://www.climb-support.org/
- TAMBA – Twins and Multiple Births Association http://www.tamba.org.uk/
- SANDS – Stillbirth and Neonatal Death Society https://www.uk-sands.org/
- CONI – Care of the Next Infant http://www.lullabytrust.org.uk/coni
- Préma-Québec www.premaquebec.ca

www.neonatalbutterflyproject.org

SUPPLEMENTAL MATERIAL:
course material in French

CHU Sainte-Justine
Le centre hospitalier universitaire mère-enfant

Université de Montréal

RQSPAL
RÉSEAU QUÉBÉCOIS DE RECHERCHE EN SOINS PALLIATIFS ET DE FIN DE VIE

Projet Ruban:

Deuil périnatal lors d'une grossesse multiple

Alexie Bigras-Mercier, inf., Sophie Bélanger, IPSNN, Audrey Larone-Juneau, Annie Janvier, MD PhD

Inspiré de "The butterfly project", par Nick Embleton

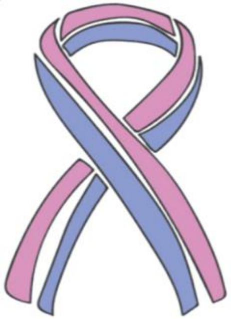

Introduction

- Jumeaux et triplés: un nombre significatif de nos admissions
- Les soignants sont souvent les seuls à avoir connu le bébé
- Coexistence de sentiments contradictoires chez les parents
- Manque de confiance du personnel dans cette situation delicate
    - Souvent ne pas le savoir et faire du tort “*avez-vous d’autres enfants?*”
    - En parler ou pas, comment?
    - Éviter les commentaires blessants “*au moins il vous en reste un* ”

Thèmes identifies importants pour les parents:

Richards et al. (2015). Mother's perspectives on the perinatal loss of a co-twin: a qualitative study. BMC Pregnancy and Childbirth, 15:143.

1. Reconnaître le statut de jumeau
2. Reconnaître le deuil
3. Offrir un soutien émotionnel
4. Fournir l'information pertinente
5. Assurer la continuité des soins
6. Confectionner des souvenirs
7. Répartition des patients sur l'unité
8. Préparer les parents au congé

1. Reconnaître le statut de jumeau

«Ils (le personnel) savaient tout ce qui c'était passé, et je crois que c'est vraiment important… personne n'a jamais essayé de nous traiter comme si on avait seulement eu un enfant» [Mère]

«… un des infirmiers… lorsqu'on était sur l'unité, a mentionné le jumeau décédé… et il nous a dit «nous savons ce qui est arrivé. Si vous voulez un jour en parler ou quoique ce soit, nous sommes là pour vous» [Mère]

Le ruban comme symbole

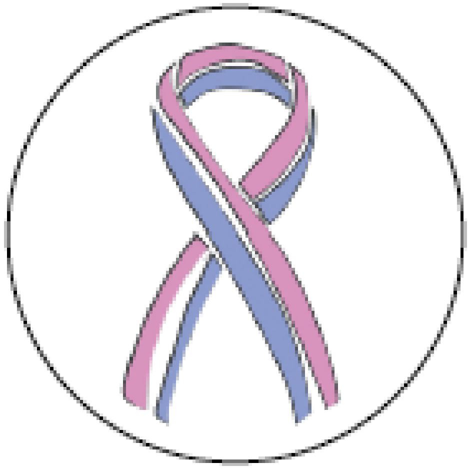

«Ils oubliaient qu'il était un jumeau» [Père] «Ce n'est pas qu'ils oubliaient, souvent ils ne le savaient simplement pas» [Mère]

«Un petit papillon bleu… un simple papillon sur l'incubateur puis ils savaient qu'il était un jumeau et le problème était réglé» [Mère]

Parents dans notre unite préfèrent le ruban symbole du deuil perinatal

Le ruban comme symbole

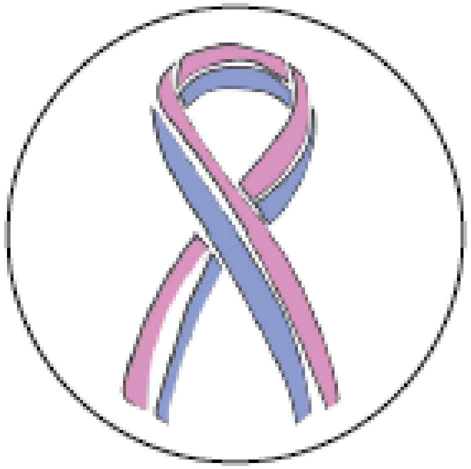

Choisit à la place du papillon (certains parents n’aimaient pas ce symbole)

Décision de placer ce symbole partout quand il y a un décès de jumeau en prénatal ou post natal:
Consultations de néonatologie
Carte du bébé
Dossier du bébé vivant
Incubateur
Plan de soins

2. Reconnaître le deuil

- Les professionnels de la santé et certains parents ont avoué que souvent, ils se concentrent trop rapidement sur le jumeau survivant.
- « ...*Nous nous concentrons sur le positif et non le deuil. Nous devrions cependant gérer la joie et le deuil en même temps» [Sage-femme]*

3. Offrir un soutien émotionnel

« Ils (le personnel) ne s'autorisaient pas le droit d'avoir de la peine autour de nous... on le ressentait lorsqu'ils quittaient la salle... c'était réconfortant de savoir qu'on n'était pas seuls à être bouleversés...» [Mère]

Les parents apprécient lorsque le personnel soignant démontre de la compassion:

«Je vais toujours être heureuse de parler de [nom du bébé décédé] avec vous. Aujourd'hui n'est peut-être pas opportun pour vous d'en parler, mais on peut toujours discuter une autre journée.»

Fournir du support moral

- Organiser le suivi avec psychologie, travail social et soins spirituels
- Écouter, écouter, écouter
- Normaliser les émotions

«C’est normal de ressentir du bonheur et de la tristesse en même temps. »

4. Fournir l’information pertinente

- Possibilité et pertinence de la proximité entre jumeaux en pré et post natal
- Qu’est-ce qui arrive après le décès
- Emplacement du corps du bébé décédé
- Groupes de soutien et aide psychologique

Connaissez-vous l'armoire du deuil sur notre unité?

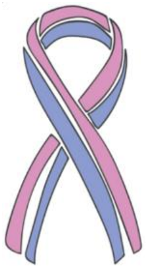

5. Assurer la continuité des soins

«C'était vraiment bien, nous connaissions (une soignante de l'équipe) et nous lui faisions confiance pour ce genre de choses et l'équipe est allée la chercher rapidement...» [Mère]

«C'était souvent une nouvelle infirmière, donc nous devions lui réexpliquer tout...» [Mère]

6. Confectionner des souvenirs

- Prendre bébé
- Photo (Portrait d'Étincelle)
- Empreintes
- Rites spirituels ou religieux

7. Répartition des patients sur l'unité

- Lorsque possible et après en avoir discuté avec les parents, déplacez le jumeau survivant dans une chambre seule et non le laisser dans la chambre de jumeau où l'autre est "remplacé"
- Discuter de tout changement avec les parents

«C'est un peu insensible de leur part de nous placer dans la même chambre que des jumeaux» [Mère]

«C'était vraiment difficile à voir, mais on ne peut pas leur demander de ne plus jamais mettre aucun autre bébé dans cet incubateur» [Mère- en parlant d'un nouveau bébé dans l'ancien incubateur de son jumeau décédé]

8. Préparer les parents pour le congé

- Organiser une rencontre pré-congé avec un membre de l’équipe soignante (médecin ou infirmière)
- Offrir du soutien au deuil (psychologie, travail social)
- Mettre les parents en contact avec un groupe de soutien au deuil
- Donner les coordonnées des organismes pouvant les aider
- S’assurer qu’une note informative soit au dossier du bébé survivant (avec le nom du bébé décédé)
- Organiser une rencontre de suivi pour les parents avec un professionnel
- Fournir des explications rassurantes sur la santé de leur jumeau survivant

Préparer les parents pour le congé

- S’assurer que les parents ont un suivi de deuil approprié
- S’assurer que le CLSC et le pédiatre au suivi sont au courant de leur deuil et du bébé décédé
  - Leur indiquer sur les requêtes et dans le résumé d’hospitalisation
  - Leur transmettre le nom du bébé décédé

Autres interventions pertinentes

- Si le bébé porte un chiffre (BB Tremblay 1 ou 2), ne pas enlever automatiquement ce chiffre si un des bébés décède, **demandez aux parents ce qu'ils préfèrent**
- Identifier ces situations (collant Ruban)
- Transférer l'information d'un quart de travail à l'autre pour éviter de poser des questions blessantes

En résumé,

- Connaître le prénom du bébé décédé et l'utiliser
- Ne pas éviter de parler du jumeau
- Ne jamais dire: «*au moins il vous en reste un*»
- Considérer la proximité des jumeaux avant le décès ou après
- Confectionner des souvenirs
- Discuter des procédures attendues en pré et post natal

Ressources

- Préma-Québec www.premaquebec.ca
- Plusieurs organismes dans chaque région: Cartable “Ressources en deuil périnatal” (armoire deuil)
- Ouvrages en deuil périnatal: Cartable dans l’armoire de deuil

- MBF – The Multiple Birth Foundation http://www.multiplebirths.org.uk/
- CLIMB – Center for Loss in Multiple Birth http://www.climb-support.org/
- TAMBA – Twins and Multiple Births Association http://www.tamba.org.uk/
- SANDS – Stillbirth and Neonatal Death Society https://www.uk-sands.org/
- CONI – Care of the Next Infant http://www.lullabytrust.org.uk/coni

www.neonatalbutterflyproject.org

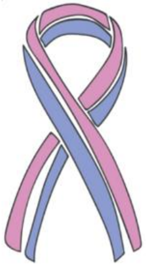

Supplement: Supplementary file 1 [file children-10-01407-s001.zip › children-2492615-supplementary.pdf]
